# Can plitidepsin be used as an antiviral against RSV?

**DOI:** 10.1128/msphere.00127-25

**Published:** 2025-11-24

**Authors:** Charlotte Estampes, Jenna Fix, Julien Sourimant, Priscila Sutto-Ortiz, Charles-Adrien Richard, Etienne Decroly, Marie Galloux, Jean-François Eléouët

**Affiliations:** 1 Unité de Virologie et Immunologie Moléculaires (VIM), INRAE, Université Paris Saclay 543420, Jouy-en-Josas, France; 2 Aix Marseille Université, CNRS, AFMB UMR 7257, Marseille, France; The University of Texas Southwestern Medical Center, Dallas, Texas, USA

**Keywords:** respiratory syncytial virus, plitidepsin, eEF1A, inhibition

## Abstract

Human respiratory syncytial virus (HRSV) is a main cause of acute lower respiratory tract infections in infants, the elderly, and immunocompromised patients. Although vaccines have recently been approved for the elderly and for pregnant women, there is no curative treatment for HRSV. HRSV replicates in the cytoplasm of infected cells, and transcription and replication of the viral genome depend on the viral RNA polymerase complex, which recruits cellular factors for RNA synthesis. Among them, the eukaryotic translation elongation factor 1A (eEF1A) was previously shown to be critical for HRSV replication. eEF1A activity can be inhibited by plitidepsin (Aplidin), a cyclopeptide extracted from the ascidian Aplidium albicans, which was shown to be highly potent against SARS-CoV-2, with a 50% inhibitory concentration (IC_90_) of 0.70 to 1.62 nM depending on the cell line. Here, we investigated whether plitidepsin could also inhibit HRSV replication. We found that plitidepsin inhibited HRSV replication with an IC_50_ of ≈3 nM in cell cultures. However, further investigation revealed that plitidepsin has pleiotropic effects, affecting the translation of both cellular and viral proteins in a similar manner. Overall, our results show that plitidepsin blocks cellular translation and indicate that plitidepsin can induce a proteasome-mediated degradation of eEF1A, depending on the cell line, also showing the dependence of HRSV replication on cellular factors, such as eEF1A. These results thus highlight an original mechanism of action of plitidepsin on eEF1A, which renders the use of this compound for antiviral therapy very risky.

**IMPORTANCE:**

Respiratory syncytial virus (RSV) is the main cause of bronchiolitis in infants and the elderly. Although some recent advances have been made, in particular vaccines for pregnant women and the elderly, or a new and efficient monoclonal prophylactic antibody for newborns, there is no curative treatment for human respiratory syncytial virus (HRSV). Previous works suggested that a natural compound extracted from a marine organism, plitidepsin, was capable of inhibiting virus replication, in particular SARS-CoV-2. Because the target of plitidepsin has been identified as the cellular protein eukaryotic translation elongation factor 1A (eEF1A) that brings tRNA-aa to the ribosome, and because it was published that RSV needs eEF1A, we tested plitidepsin against RSV. During this work, by using a non-radioactive pulse-chase labeling of protein synthesis, we found that plitidepsin blocks cellular translation with no specificity for the virus. We also observed that eEF1A was degraded after plitidepsin treatment in the BHK21-derived BSRT7 cell line, and that this degradation was inhibited by a proteasome inhibitor. However, this was not observed with Human HEp-2 or simian Vero E6 cell lines. So, we think that our results are new and original and that this information should be useful for the community working either with plitidepsin or eEF1A, with viruses, or other topics. We think that, in contrast to what is suggested by previous studies, it is risky to use plitidepsin as an antiviral in humans.

## INTRODUCTION

Human respiratory syncytial virus (HRSV) is the commonest cause of lower respiratory tract infection in young children worldwide and the first cause of their hospitalization (1). HRSV is estimated to infect about 33 million children, leading to more than 3 million hospitalizations and 26,000–152,000 deaths in children under 5 years each year (2). The global healthcare costs of HRSV-associated infections in young children in 2017 were estimated to be US$5.45 billion (3). In a systemic multisite study, HRSV was shown to be the first etiological agent responsible for severe pneumonia (more than 30%) in hospitalized children in Asia and Africa (4). HRSV infections are also associated with significant morbidity and mortality in the elderly and immunocompromised people (5, 6). The true burden of disease in adults is likely significantly under-recognized, and recent studies indicate that the HRSV impact is similar to that of seasonal influenza in adults older than 65 years (7–12).

HRSV vaccine research has been ongoing for nearly 60 years without success, and only in 2023, two vaccines based on stabilized HRSV prefusion F protein, Arexvy (GSK) and Abrysvo (Pfizer), were approved for medical use for adults aged 60 or older, both in the USA and Europe. In September 2023, Abrysvo was also approved in the USA for pregnant women at 32–36 weeks’ gestation to prevent HRSV infection in newborn children. Finally, Moderna received U.S. FDA and E.U. approval for RSV Vaccine mRESVIA (13). The only pharmaceutical intervention since 1998 has been passive prophylaxis with Palivizumab, a monoclonal antibody targeting the fusion protein, thereby limiting the HRSV entry. However, its use was limited to high-risk infants because of the elevated cost and moderate efficacy (14). But recently, nirsevimab (Beyfortus), a long-acting monoclonal antibody, was approved by several regulatory agencies around the world for the prevention of HRSV infections in newborns and infants (15). A single intramuscular injection of this antibody should protect infants for an entire season compared with monthly doses and reduce costs (vaccine-like pricing expected), allowing for administration to all infants. However, there is still no specific curative treatment against HRSV.

HRSV is a non-segmented single-stranded negative-sense RNA virus of the *Mononegavirales* order, *Pneumoviridae* family, and *Orthopneumovirus* genus (16). The HRSV genome is approximately 15.2 kb long and contains 10 genes encoding 11 proteins (17). Replication and transcription rely on four of these proteins: the nucleoprotein N involved in genome and antigenome encapsidation, forming the ribonucleoprotein complex (NC), the RNA-dependent RNA polymerase L which exhibits all the enzymatic activities required for viral replication, transcription, and RNA capping, its cofactor the phosphoprotein P responsible for recruitment of L on the NC template, and the transcription factor M2-1 that has been described as an “antiterminating” factor during transcription and interacts with P and viral mRNAs (18). All viral RNA synthesis takes place in viral factories also called cytoplasmic inclusion bodies (19), which concentrate the viral proteins L, N, P, and M2-1, but also cellular proteins, such as the protein phosphatase 1 (PP1), HSP70, or the human eukaryotic translation elongation factor 1A (eEF1A), all involved in HRVS replication (20–22). Interestingly, it was found that many RNA viruses utilize eEF1A for replication, although the mechanisms by which they do this differ (23). In a previous study in which eEF1A was knocked down or inhibited with didemnin B, it was suggested that eEF1A plays a key role in the regulation of F-actin stress fiber formation required for HRSV assembly and release, but with no effect on HRSV genome replication (24). Furthermore, eEF1A was found to be associated with the N and P proteins in infected cells by using a proximity ligation assay and co-immunoprecipitation (22). Recently, plitidepsin (Aplidin), an analog of didemnin B and a potent anti-cancer agent targeting eEF1A2 (K_D_ = 80 nM) (25), was shown to be highly potent against SARS-CoV-2 by targeting eEF1A, with a 90% inhibitory concentration (IC_90_) of 0.88 nM (26). As suggested by the authors, since HRSV also uses eEF1A for viral replication, inhibition of eEF1A could be a new strategy to limit HRSV propagation/infection. In this work, we thus investigated the effect of plitidepsin on HRSV replication. We show that plitidepsin inhibits HRSV replication in infected cells, as well as a minigenome reporter system with an IC_50_ of ≈3 nM in cultured cells. Further mechanistic investigation revealed that plitidepsin can induce the degradation of eEF1A in the BHK21-derived cell line, inhibiting the translation of both viral and cellular proteins in a similar range of concentration.

## MATERIALS AND METHODS

### Cells

BSRT7/5 cells (BHK-21 cells that constitutively express the T7 RNA polymerase) (27) and HEp-2 cells (ATCC: CCL-23) were maintained, respectively, in DMEM and MEM supplemented with 10% heat-inactivated fetal calf serum (FCS), with 2 mM glutamine, 100 µg/mL penicillin, and 100 U/mL streptomycin. Vero E6 cells were cultivated as BSRT7/5 cells. Cells were grown in an incubator at 37°C in 5% CO_2_. Transfections were performed with 2.5 µL of Lipofectamine 2000 (Thermofisher) per 1 µg of DNA according to the manufacturer’s instructions.

### Viruses

Recombinant human RSV rHRSV-mCherry corresponding to HRSV Long strain expressing the mCherry protein was amplified on HEp-2 cells and titrated using a plaque assay procedure as previously described (28, 29).

### Plitidepsin antiviral activity

Plitidepsin (MedChemExpress) and Carfilzomib (Cell Signaling #15022) were solubilized in DMSO at 1 and 5 mM as stock solutions, respectively. HEp-2 cells in 96-well plates were infected for 2 h with rHRSV-mCherry at an MOI of 0.2, in the absence of FCS. The medium was then changed to the same medium with 2% FCS and containing serial dilution of plitidepsin, with a 1% final concentration of DMSO in the culture medium. At 48 h post-infection, the red fluorescence intensity of mCherry was quantified using a Tecan infinite M200Pro spectrofluorometer with excitation and emission wavelengths of 580 and 620 nm, respectively. Values obtained for non-treated infected and non-infected cells were used for standardization and normalization. In parallel, the toxicity of the treatment was assessed on non-infected cells using the CellTiter-Glo Luminescent cell viability assay (Promega). The half-maximal inhibitory concentration (IC_50_) and cytotoxic concentration (CC_50_) were determined by fitting the data to the dose-response curve implemented in GraphPad version 8 software.

### Fluorescence microscopy

HEp-2 cells infected with rHRSV-mCherry were fixed with PBS-paraformaldehyde 4% for 20 min at room temperature, rinsed with PBS, and permeabilized with PBS-BSA 1%—Triton X-100 0.1% for 10 min. Nuclei were stained with Hoechst 33342 (1 µg/mL) for 5 min, washed with PBS, examined under a Nikon TE200 microscope equipped with a CoolSNAP ES2 (Photometrics) camera, and images were processed with Meta-Vue software (Molecular Devices).

### Minigenome assay

BSRT7/5 cells at 90% confluence in 96-well dishes were transfected with a plasmid mixture containing 125 ng of pM/Luc, 125 ng of pN, 125 ng of pP, 62.5 ng of pL, and 31 ng of pM2-1, as well as 31 ng of pRSV-β-Gal (Promega) to normalize transfection efficiencies as described previously (30, 31). After 6 h, the transfection mix was removed and serial dilutions of plitidepsin were added for 14 h. Transfections were done in triplicate, and each independent transfection was performed three times. Cells were harvested 24 h post-transfection, then lysed in luciferase lysis buffer (30 mM Tris, pH 7.9, 10 mM MgCl_2_, 1 mM DTT, 1% Triton X-100, and 15% glycerol). The luciferase and β-galactosidase (β-Gal) activities were determined for each cell lysate with an Infinite 200 Pro (Tecan, Männedorf, Switzerland) and normalized based on the values obtained for cells treated with DMSO only.

### Fluorescence-based nucleotide-incorporation assay

The analysis of the effect of Plitidepsin on the RSV polymerase activity was performed according to the method described by Sutto et al. (32). The RdRp assay reaction contained 0.2 µM recombinant RSV L-P, 2 µM of oligonucleotide template, and 2 µM of a 5′-6-FAM primer, mixed in a buffer containing 20 mM Tris-HCl, pH 7.5, 10 mM KCl, 2 mM DTT, 0.01% Triton X-100, 5% DMSO, 0.2 U/mL RNasin (Ambion), and 6 mM MgCl_2_. Reactions were started by adding specific NTPs at 100 µM and incubated for 2 h at 30°C. Reactions were quenched by adding an equal volume of gel-loading buffer. Samples were denatured at 70°C for 10 min and run on a 20% polyacrylamide urea sequencing gel for 2.5 h at 45 W. The gel was scanned on a Typhoon imager (GE Healthcare).

### Western blotting

Cells were lysed in 2× Laemmli buffer, run on a 12% polyacrylamide gel, and transferred to a nitrocellulose membrane using the Trans-Blot Turbo system (Bio-Rad). The blots were blocked with 5% nonfat milk in PBS Tween20 0.2%, and probed with either a polyclonal rabbit anti-N serum (33), a rabbit anti-GFP (Invitrogen, Waltham, MA, USA), a mouse monoclonal anti-EF1A antibody (Santa Cruz G-8, sc377439), a mouse monoclonal anti-GAPDH antibody (Sigma-Aldrich MAB374), or a mouse anti-alpha-tubulin (DM1A, Sigma) and further incubated with HRP-coupled anti-mouse or anti-rabbit antibodies (Life Science). Membranes were revealed with the Clarity Western ECL kit (Bio-Rad) and analyzed with a ChemiDoc Touch Imaging System and Image Lab software (Bio-Rad).

### Pulse labeling of newly synthesized proteins with L-azidohomoalanine

HEp-2 cells were infected with rHRSV-mCherry at MOI = 1 in 6-well plates (35 mm in diameter) and incubated in MEM supplemented with 2% heat-inactivated FCS, 2 mM glutamine, 100 µg/mL penicillin, and 100 U/mL streptomycin. Twenty-four hours later, serial dilutions of plitidepsin or DMSO were added to the cells for 2 h. Cells were washed twice with methionine-free (Met-) MEM (Gibco), then live-labeled (pulsed) at 37°C for 4 h with 50 µM Click-iT L-azidohomoalanine (AHA; Jena Bioscience) in Met-MEM supplemented with 5%–10% dialyzed FBS (Gibco) and plitidepsin. Cells were lysed in 1 mL of RIPA buffer (50 mM Tris-HCl [pH 8.0], 150 mM NaCl, 1% Triton X-100, 2 mM DTT, 10 mM of freshly prepared iodoacetamide [Merck], and complete protease inhibitor cocktail [Roche]). After centrifugation for 10 min at 10,000 rpm at 4°C, the supernatants were mixed with 5 µM AFDye 488-DBCO (Click Chemistry Tools, Jena Bioscience) and incubated for 2 h at 21°C. Proteins were immunoprecipitated with specific antibodies coupled to protein A sepharose beads (GE Healthcare) and analyzed by SDS-PAGE, together with the supernatants. Fluorescence was analyzed with a Fuji FLA-3000 scanner and quantified using the AIDA or Fiji software.

## RESULTS

### Antiviral potency of plitidepsin against HRSV

**Fig 1:**
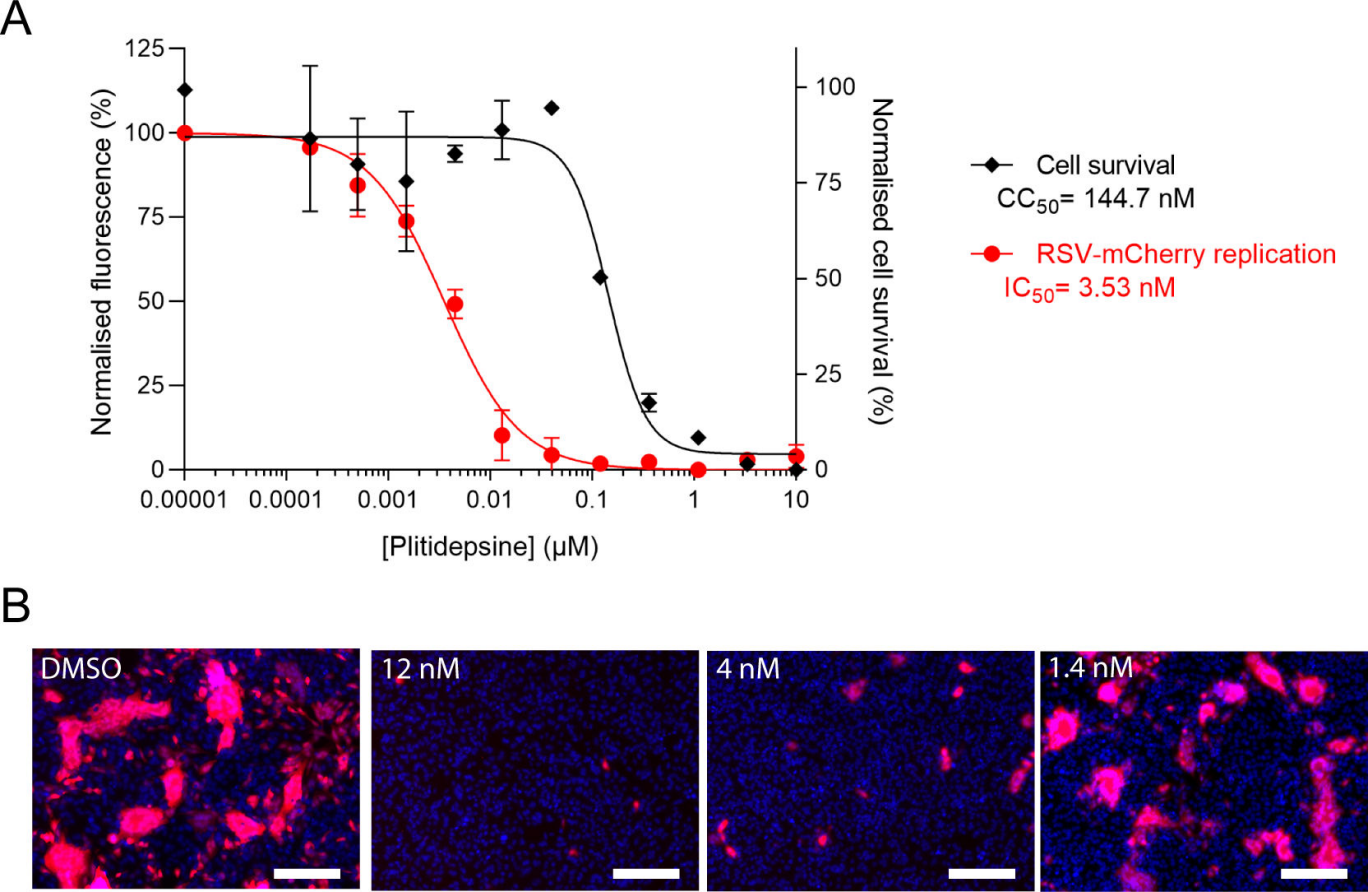
Impact of plitidepsin treatment on HRSV replication on HEp-2 cells. (**A**) Cells were infected for 2 h with rHRSV-mCherry at MOI 0.2, and the medium was then replaced to incubate cells in the presence of serial dilutions of plitidepsin for 48 h (red curve). The viral replication was quantified by measurement of the mCherry fluorescence. In parallel, cell viability upon treatment with plitidepsin was quantified in non-infected HEp-2 cells (black curve). Error bars are standard deviations from duplicates. Data are representative of three experiments. The curves were fitted in GraphPad 8 software using a four-parameter logistic regression. Both IC_50_ and CC_50_ are indicated. (**B**) Representative images of HEp-2 cell cultures infected with rHRSV-mCherry at MOI 0.2, 48 h post-infection in the absence or presence of plitidepsin at 12, 4, and 1.4 nM. Nuclei were colored with Hoechst 33432. Scale bars, 250 µm.

By using an siRNA approach, it was demonstrated 10 years ago that downregulation of eEF1A correlates with a reduction in the amount of infectious HRSV release, accompanied by a reduction in viral genome expression, but not mRNA transcription or protein expression (22). We therefore tested whether plitidepsin, known to inhibit the eEF1A activity, displays antiviral activity against HRSV. For this purpose, HEp-2 cells were infected with rHRSV-mCherry at MOI = 0.2 in a culture medium containing increasing concentrations of plitidepsin. Viral replication was quantified by mCherry fluorescence measurement on live cells 48 h post-infection using the Tecan spectrofluorometer. Values were standardized and normalized by those obtained for non-treated infected and non-infected cells. The IC_50_ was determined by dose-response curve fitting. In parallel, the toxicity of plitidepsin was evaluated on non-infected HEp-2 cells. As shown in Fig. 1A, plitidepsin inhibited rHRSV-mCherry replication in a dose-dependent manner, with an IC_50_ of 3.5 nM. These data were confirmed by direct observation of mCherry fluorescence and nuclei staining on fixed rHRSV-mCherry-infected cells (Fig. 1B). Noteworthy, a cytotoxic activity of plitidepsin was observed, with a calculated half-maximal cytotoxic concentration (CC_50_) close to 145 nM (Fig. 1A). With a calculated selective index >30, plitidepsin thus appeared to be a potent inhibitor of HRSV replication in HEp-2 cells.

In a recent paper, using Vero E6 cells, Molina Molina et al. (34) found an inhibitory effect of plitidepsin against HRSV with an IC_50_ of 27 nM, thus five times higher than the one we found using HEp-2 or BSRT7 cells and the IC_50_ they measured for SARS-CoV-2 inhibition. The authors hypothesized that plitidepsin promotes a shift in mRNA translation from a cap-dependent mechanism to a cap-independent pathway promoted by m6A mRNA methylation. Consistent with this hypothesis, they concluded that the stronger inhibitory effect of plitidepsin on SARS-CoV-2 replication results from the suppression of cap-dependent mRNA translation, leading to a more pronounced reduction in viral mRNA abundance and viral protein synthesis compared to host mRNA translation and cellular protein production. Conversely, the weaker effect observed on RSV was attributed to the virus’s ability to exploit m6A-dependent translation pathways, as previously reported for RSV (34). To further validate this hypothesis, we evaluated the antiviral activity of plitidepsin against RSV in Vero E6 cells using the same experimental conditions as those employed for HEp-2 cells. However, as shown in Fig. S1, in our hands, plitidepsin also inhibited rHRSV-mCherry replication in a dose-dependent manner, but with an IC_50_ of ~5 nM, thus in the same range as the one measured when using HEp-2 cells.

### Effect of plitidepsin on an HRSV minigenome

**Fig 2:**
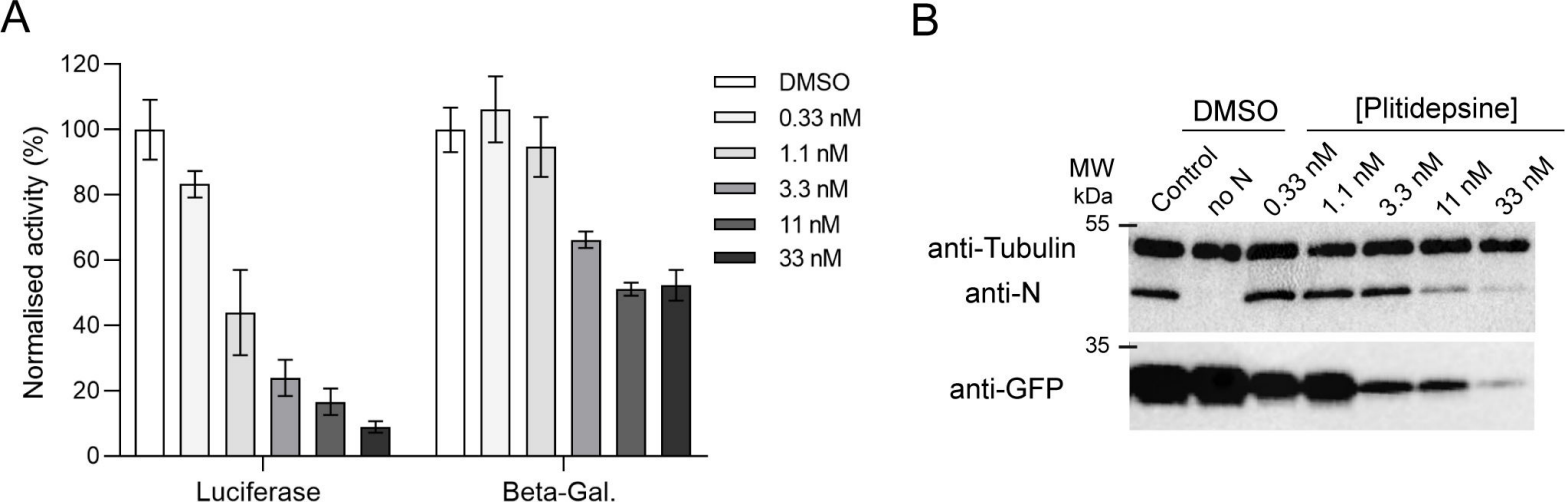
Plitidepsin effect on an HRSV minigenome. (**A**) BSRT7/5 cells were transfected with plasmids encoding the N, P, M2-1, and L proteins; the pMT/Luc minigenome, together with pCMV-beta-gal for transfection standardization. Serial dilutions of plitidepsin were added 6 h post-transfection after removing transfection reagents. Viral RNA synthesis was quantified by measuring the luciferase activity after cell lysis 24 h post-transfection. Each luciferase minigenome activity value was normalized based on DMSO-treated cells and is the average of three independent experiments performed in triplicate. Error bars represent standard deviations calculated based on three independent experiments made in triplicate. (**B**) BSRT7 cells were transfected with the complete minigenome system (see above) or with the pEGFP plasmid coding for EGFP. Six hours later, the transfection mix was removed, and medium containing plitidepsin at different concentrations was added. Then, 24 h post-transfection, cells were lysed and analyzed by western blotting using anti-N, anti-GFP, or anti-alpha tubulin antibodies.

To determine whether plitidepsin could affect the RNA polymerase replication/transcription machinery of HRSV, we used a well-established HRSV-specific minigenome assay system (30). The pM/Luc plasmid, which contains the authentic M/SH gene junction and the Luc reporter gene downstream of the gene start sequence inserted in this gene junction, was co-transfected into BSR-T7/5 cells together with p-β-gal, pL, pP, PN, and pM2-1. Luciferase activity was determined and normalized based on the signal obtained for cells incubated in a similar medium with 1% DMSO. In parallel, β-galactosidase (β-Gal) activity was determined for each cell lysate in order to normalize transfection efficiency. Figure 2A shows that plitidepsin inhibits the function of the HRSV polymerase complex in a dose-dependent manner with an IC_50_ of ~5 nM while showing no toxicity toward BSR-T7/5 cells. However, plitidepsin also inhibited, to a lesser extent, the β-Gal activity, the effect being visible from 3.3 nM of plitidepsin.

Since plitidepsin targets the eEF1A cellular translation factor, the inhibition of HRSV RNA polymerase activity could result from either the direct inhibition of polymerase activity of the HRSV L protein or from the inhibition of translation of the viral mRNAs coding for the viral proteins. To assess whether the plitidepsin treatment had a specific or global impact on protein expression, cells were co-transfected either with the minigenome system or with a plasmid encoding EGFP, in the presence of increasing concentrations of plitidepsin. The expression of proteins was analyzed by western blot. As shown in Fig. 2B, a similar decrease in N and EGFP expression was observed in the presence of plitidepsin, indicating that plitidepsin had a general inhibitory effect on viral or non-viral protein expression. In contrast, the signal corresponding to alpha-tubulin did not decrease significantly, which can easily be explained by the half-life of this cellular protein (~8 days) (35).

### Effect of plitidepsin on *in vitro* HRSV RdRp activity

**Fig 3:**
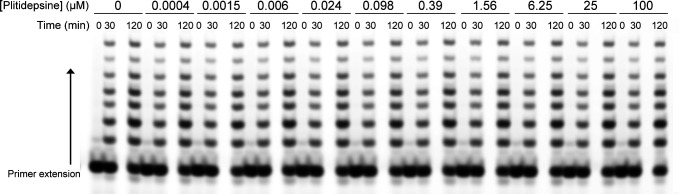
*In vitro* effect of plitidepsin on HRSV RNA polymerase (L-P complex) activity. Primer extension polymerase assay was performed in the presence of increasing concentration of plitidepsin. Briefly, 200 nM of RSV L/P was incubated with 2 µM of 11-nucleotide-length RNA template and four-nucleotide primer in the presence of increasing concentrations of plitidepsin. The reactions were incubated for 30 and 120 min at 30°C, and the reaction products were separated on a 20% UREA-PAGE gel before gel scanning on a Typhoon imager.

To test whether plitidepsin could also directly affect the HRSV RNA polymerase activity, we performed an *in vitro* RNA polymerase assay using a recombinant L-P complex (36–38) in the presence of increasing plitidepsin concentrations. As shown in Fig. 3, no inhibitory effect of plitidepsin on RNA synthesis was observed, even for a concentration of 100 µM of plitidepsin. These results strongly suggest that the effect of plitidepsin on viral replication was not due to a direct effect of the RdRp complex but mainly due to its impact on protein expression.

### Effect of plitidepsin on cellular and viral protein neosynthesis

**Fig 4:**
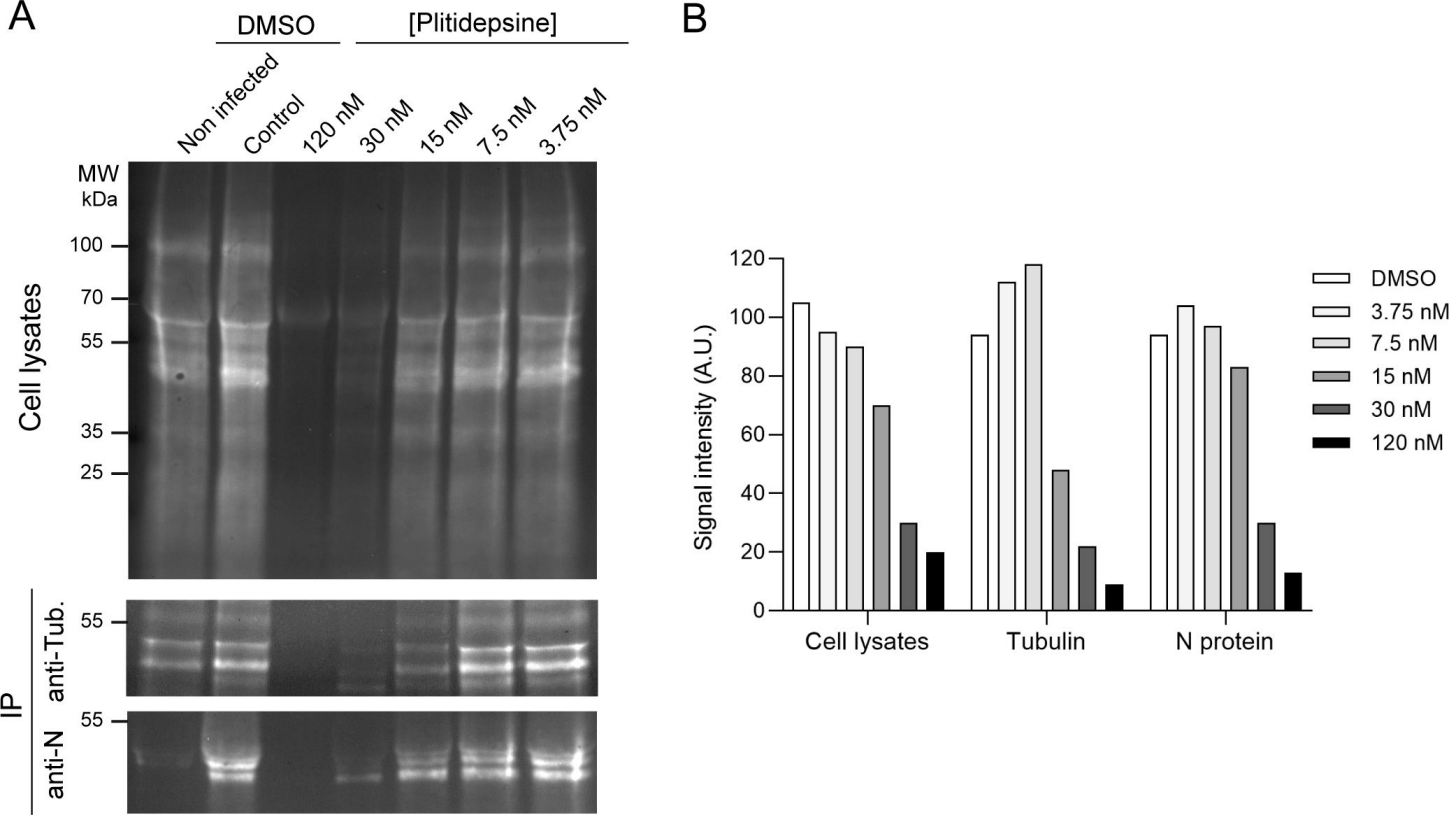
Compared effect of plitidepsin on viral or cellular protein synthesis. HEp-2 cells in 6-well plates were infected with rHRSV-mCherry at MOI 1. Twenty-four hours later, the medium was changed for protein labeling with AHA with serial dilutions of plitidepsin. After 4 h, cells were lysed, and AHA-labeled proteins were revealed with AFDye 488-DBCO, then immunoprecipitated with anti-N or anti-alpha-tubulin antibodies. Immunoprecipitated proteins or supernatants were resolved by SDS-PAGE (**A**), and fluorescence in the gels was measured using a Fuji FLA-3000 scanner (**B**).

To further investigate the impact of plitidepsin on viral and cellular RNA translation, we used a complementary approach, consisting of a pulse labeling of newly synthesized proteins with the methionine analog AHA, revealed by the fluorescent dye AFDye 488-DBCO based on Click chemistry (39). At 24 h post-infection, HEp-2 cells were incubated with serial dilutions of plitidepsin and then labeled with AHA for 4 h. Then, cells were lysed, and the HRSV N protein or the cellular α-tubulin protein was immunoprecipitated. Immunoprecipitated proteins and total cell lysates were analyzed by SDS-PAGE, and the fluorescence was quantified. As shown in Fig. 4A and B, a similar drop in protein synthesis was observed for N and α-tubulin, but also for the total proteins present in the cell lysates for concentrations ≥15 nM. These results correlate with the observations made on transfected BRST7 cells and highlight that plitidepsin has a similar negative impact on the translation of both viral and cellular mRNA.

### Plitidepsin induces proteasome-mediated degradation of eEF1A in BHK21-derived cell line

**Fig 5:**
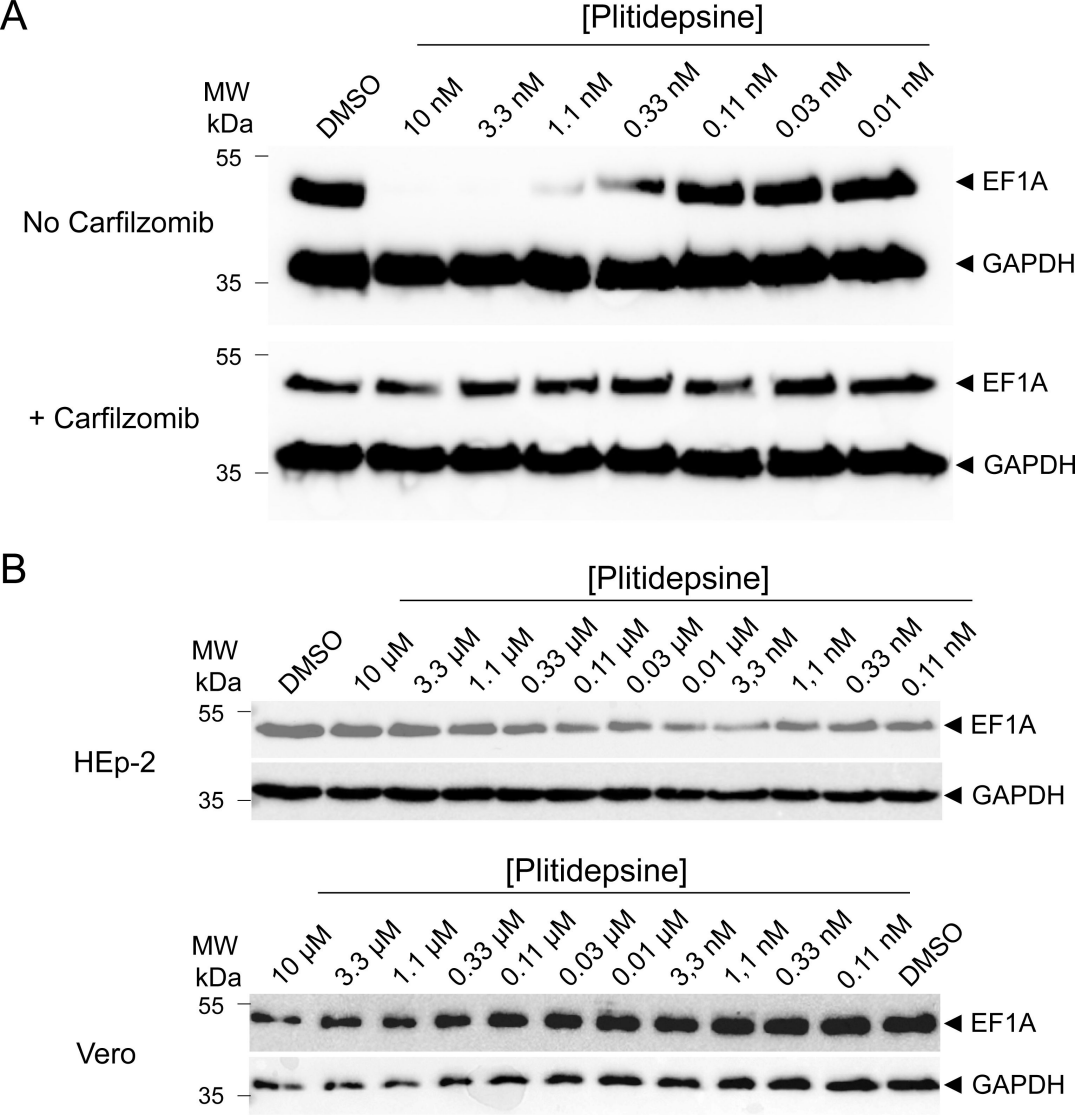
Antiviral mechanism of action of plitidepsin is mediated through proteasome-mediated degradation of eEF1A in BSRT7/5 cells. BSRT7 (**A**) or HEp2 and Vero (**B**) cells were treated with serial dilutions of plitidepsin or DMSO for 18H in the presence or not of the proteasome inhibitor Carfilzomib (500 nM), and the expression of eEF1A or GAPDH was analyzed by WB.

The mechanism by which plitidepsin inhibits the activity of the host factor eEF1A is still debated. It was shown that overexpression of a negative dominant mutant, an Ala399 → Val (A399V) of eEF1A, reduced the sensitivity of cancer cells to didemnin B (40), as well as SARS-CoV-2 to plitidepsin by a factor >10 (26). Interestingly, this A399V substitution also confers resistance to the structurally unrelated ternatin-4 (40), suggesting that these molecules interact with the same binding site on eEF1A (26). However, the mechanism of action of ternatin-4 was recently studied in more detail and revealed that it was mediated by ubiquitination and proteasome degradation of eEF1A in HeLa cells (41). We thus wondered whether plitidepsin could also induce degradation of eEF1A by a similar mechanism. The three cell lines used in this study, BSRT7, HEp-2, and Vero cells, were treated with serial dilutions of plitidepsin in the presence or absence of the proteasome inhibitor carfilzomib, and the expression of eEF1A or the control GAPDH was analyzed by western blot. As shown in Fig. 5A, for concentrations of plitidepsin inhibiting HRSV replication, minigenome activity or translation inhibition, eEF1A expression was impaired in BSRT7 cells, and expression of eEF1A was restored upon treatment in the presence of the proteasome inhibitor Carfilzomib. Of note, this effect was clearly visible as soon as 4–5 h post-treatment (Fig. S2). By contrast, no degradation of eEF1A was observed in HEp-2 and Vero cells (Fig. 5B). These results suggest that plitidepsin, in addition to inhibiting cellular translation, can also induce the degradation of eEF1A by ubiquitination and proteasome degradation, but depending on the cell line.

## DISCUSSION

The mammalian translation elongation factor eEF1A is an essential GTPase and the second most abundant intracellular protein after actin (3% of the total cellular protein). It is localized extensively in the cytoplasm and nucleus (23, 42, 43). The canonical function of eEF1A is to deliver aminoacyl tRNAs to the ribosomal A site during the elongation stage of protein synthesis. In addition to its canonical functions in transporting aa-tRNA to the ribosome, eEF1A is found to be involved in cellular mechanisms, such as regulation of cytoskeleton organization by interacting with actin and tubulin, protein degradation mediated by the proteasome, nuclear aa-tRNAs and protein export, signaling transduction pathway concerning apoptosis and oncogenesis, or binding to viral RNA (43–45). There are two variants of eEF1A, eEF1A1 and eEF1A2, that share 92% amino acid identity (46). In contrast to the ubiquitous expression of eEF1A1 in many cell types, eEF1A2 expression is limited to the terminally differentiated cells of the brain, heart, and skeletal muscle (47). The two eEF1A variants have similar translation activity but may differ with respect to their secondary, “moonlighting” functions. eEF1A1 also plays an important role in the process of heat shock stress response. eEF1A2 activates Akt in a PI3K-dependent fashion, stimulating cell migration, actin remodeling, and invasion, and inhibiting apoptosis (23).

Like for many viruses (23, 43), eEF1A has been identified as an important cellular factor for HRSV replication (22). As an abundant, multifunctional protein, it is not surprising that many viruses have adapted to use eEF1A as a cofactor for viral transcription, translation, assembly, and pathogenesis. So, eEF1A was shown to bind to some viral RNA structures, some viral structural and non-structural proteins, or to interact with viral polymerase complexes, such as those of vesicular stomatitis virus (VSV) (43, 48). Like for HRSV, VSV forms two different viral RNA polymerase complexes in infected cells: the transcriptase and the replicase. The transcriptase complex synthesizes capped mRNAs, whereas the replicase complex initiates genomic minus-strand RNA synthesis at the precise 3′ ends of the plus-strand antigenomic and negative-strand genomic RNAs. The transcriptase was described as a protein complex containing the L and P proteins, the cellular eEF1A and heat shock protein 60, and a submolar amount of cellular mRNA cap guanylyltransferase (49). The replicase complex was described as containing the viral proteins L, P, and nucleocapsid (N) but not eEF1A, heat shock protein 60, or the guanylyltransferase. Hence, it was proposed that eEF1A is important for transcription of viral mRNAs but not for genomic RNA replication in VSV.

Disentangling the canonical and non-canonical roles of eEF1A and the extent to which each contributes to viral function is essential if eEF1A is to be targeted therapeutically. It was previously shown that didemnin B treatment, a drug targeting eEF1A, protected cells from HRSV-induced cell death (22). Didemnin B did not significantly affect HRSV transcription and replication, especially at 24 h post-infection, but significantly reduced infectious virus production and release, possibly as a consequence of changes in actin stress fiber formation (24). More recently, plitidepsin (Aplidin), an analog of didemnin B that was approved in Australia for the treatment of myeloma (50), was shown to be very efficient against SARS-CoV-2 in cultured cells, as well as in a mice model (26). Since it has entered clinical trials as an anti-SARS-CoV-2 drug and was shown to have a favorable long-term safety profile in adult patients hospitalized for COVID-19 (51–54), we wondered whether this compound could also be efficient against HRSV. Plitidepsin is a cyclic depsipeptide that was first isolated from a Mediterranean marine tunicate (*Aplidium albicans*) and, at present, is manufactured by total synthesis and commercialized by PharmaMar, S.A., as Aplidin (55). Plitidepsin has antitumoral and immunosuppressive activities (56). Although not fully clarified, the molecular mechanisms of action of plitidepsin against tumor cells and SARS-CoV-2 have been investigated. Plitidepsin induces cell-cycle arrest and apoptosis (57). These effects rely on the induction of early oxidative stress, the rapid activation of Rac1 GTPase, and activation of c-Jun N-terminal kinase (JNK), ERK, and p38 mitogen-activated protein kinases (p38/MAPK), which finally result in caspase-dependent apoptosis (58, 59). It was determined that eEF1A is the primary target of plitidepsin, which can bind to eEF1A at the interface between domains 1 and 2 of this protein in the GTP conformation with a measured KD of 80 nM (25). However, it was proposed that the antitumoral effect of plitidepsin is not due to translation inhibition but inhibition of eEF1A binding to double-stranded RNA-dependent protein kinase (PKR). In the presence of plitidepsin, PKR would disengage from eEF1A, thereby regaining its kinase activity to initiate extrinsic apoptosis through activation of MAPK and NF-κB signaling cascades (60). Plitidepsin was also reported as an ER stress inducer by activating the unfolded protein response (61). In parallel, plitidepsin was also shown to induce the phosphorylation of eIF2a, resulting in the arrest of protein synthesis at the initiation step (61, 62). For SARS-CoV-2, it has been determined that the effect of plitidepsin is also mediated by eEF1A by using a mutated (A399V) version of eEF1A1 in 293T cells (26). It is likely that this effect is mediated by the inhibition of translation of the viral proteins (26, 57, 63). Very recently, the mechanism of SARS-CoV-2 inhibition was revisited (34). In this paper, using Vero E6 cells, the authors found that plitidepsin at 50 nM reduced the translation of RNAs, including cellular RNAs, but with a higher impact on viral mRNA translation and without affecting cellular viability. The molecular mechanisms involved in the cell’s proteostatic response to eEF1A blockade, namely a shift from cap or internal ribosome entry sites-mediated translation toward an N-6-methyladenosine (m6A)-dependent translation, that could explain cell survival. They also tested plitidepsin against several other viruses, including HRSV, and found an IC_50_ of 27 nM in Vero E6 cells, about ten times more than what we found using HEp-2 cells. For *in vivo* effects, it was also shown that treatment of a monocyte-derived macrophage cell line by plitidepsin in the same range as ours (2.5–10 nM) reduced the production of the proinflammatory cytokine IL-6 in the presence of SARS-CoV-2 virions, and that was associated with a reduction of NF-κB p65 subunit phosphorylation and of its transcription activity in the inflammatory cascade (64). This effect was also observed *in vivo* in SARS-CoV-2 or influenza virus-infected mice.

In our experiments, we observed that plitidepsin had an inhibitory effect on viral replication and minigenome activity with an IEC_50_ ≈ 3–5 nM, and a toxic effect on cells with an IC_50_ ≈ 150 nM. However, further investigation revealed that this inhibitory effect was due to a general inhibition of translation in the host cell, with no significant difference between cellular and viral protein expression. We then wondered whether eEF1A, which is essential for cellular translation, could be actively degraded by plitidepsin treatment, as it was observed recently with ternatin-4, another eEF1A-targeting drug (41). We found that, in the BHK21-derived cell line BSRT7, eEF1A was degraded after plitidepsin treatment for 18H at concentrations as low as 1 nM, which was inhibited by the proteasome inhibitor carfilzomib, indicating that an eEF1A ubiquitination mechanism can occur. However, this effect on eEF1A was inhibited in the presence of the proteasome inhibitor carfilzomib, indicating that an eEF1A ubiquitination mechanism can occur. Strikingly, this degradation was not observed when using Vero or HEp-2 cells. During this study, it was published by Molina Molina et al. that cellular cap-dependent translation was inhibited by plitidepsin in Vero cells (34), which is in accordance with our results. They also determined that plitidepsin inhibits SARS-CoV-2 with an IC_50_ varying from 4.6 to 17.9 nM depending on the viral strains, by decreasing *de novo* cap-dependent translation of SARS-CoV-2 and non-viral RNAs but affecting less than 13% of the host proteome, thus preserving cellular viability. They compared the effect of plitidepsin on other viruses, including HRSV, and found an IC_50_ of ~27 nM. Although in the nanomolar range, their hypothesis to explain this reduced sensitivity to plitidepsin was that RNA viruses having m^6^A methylation in their mRNAs, a property described for RSV (65), would use as an alternative cap-independent translation mechanism via m^6^A reading. They also found that at 50 nM plitidepsin, cellular translation was inhibited, but the host’s protein levels were poorly affected. Using pulse-chase experiments, we found that cellular translation was mainly affected in the presence of 30 nM of plitidepsin after 6 h of treatment (see Fig. 4). A simple explanation for cell survival despite translation stoppage would be to consider the persistence of cellular functions thanks to half-lives of cellular proteins which are generally long (35, 66).

In conclusion, we show here that treatment of cells with plitidepsin induces the arrest of translation in treated cells and the possible degradation of eEF1A by the proteasome pathway, depending on the cell line, which is correlated with a global extinction of translation. These experiments also show the dependence of HRSV replication on the cellular factor eEF1A. Although effective concentrations of plitidepsin against HRSV are in the same range as those found for SARS-CoV-2, the side effects of plitidepsin on cells and its potential toxicity raise the question of its use *in vivo* for antiviral assays against HRSV.

## SUPPLEMENTAL MATERIAL

Supplemental Material msphere.00127-25-s0001.docx Figures S1 and S2.

ASM does not own the copyrights to Supplemental Material that may be linked to, or accessed through, an article. The authors have granted ASM a non-exclusive, world-wide license to publish the Supplemental Material files. Please contact the corresponding author directly for reuse.
