# Supplementary material for: Can plitidepsin be used as an antiviral against RSV?
Source: mSphere. 2025 Nov 24;10(12):e00127-25. doi: 10.1128/msphere.00127-25 (PMC12724346; doi:10.1128/msphere.00127-25)
Supplement: Supplemental Material — Figures S1 and S2. [file msphere.00127-25-s0001.docx]

**
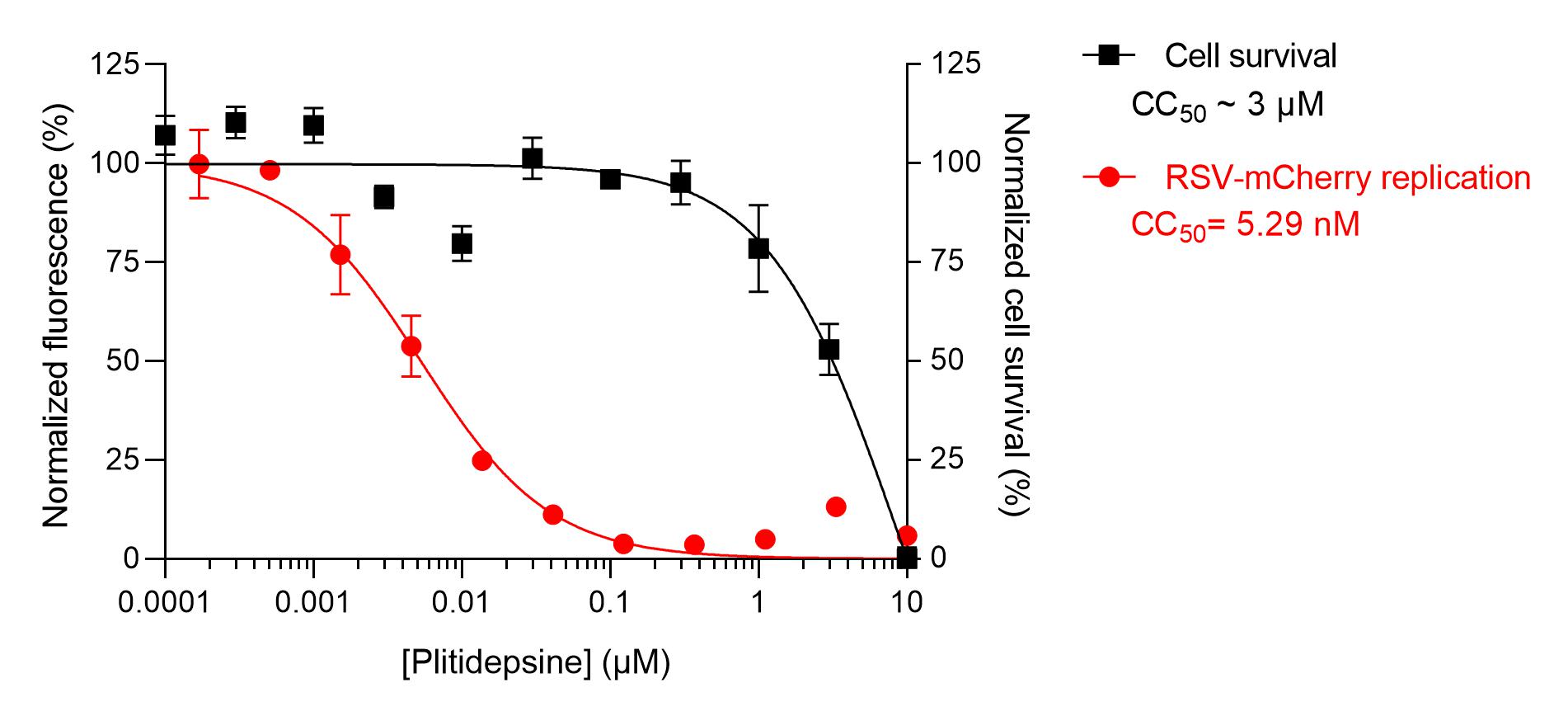
Supplementary Figure 1.** Effect of plitidepsin treatment on HRSV replication on Vero E6 cells. Cells were infected for 2 h with rHRSV-mCherry at MOI 0.2 and the medium was then replaced to incubate cells in the presence of serial dilutions of plitidepsin for 48 h (red curve). The viral replication was quantified by measurement of the mCherry fluorescence. In parallel, cell viability upon treatment with plitidepsin was quantified in non-infected cells (black curve). Error bars are standard deviations from duplicates. Data are representative of three experiments. The curves were fitted in Graph Pad 8 software using a four parameters logistic (4PL) regression. Both IC_50_ and CC_50_ are indicated.

**
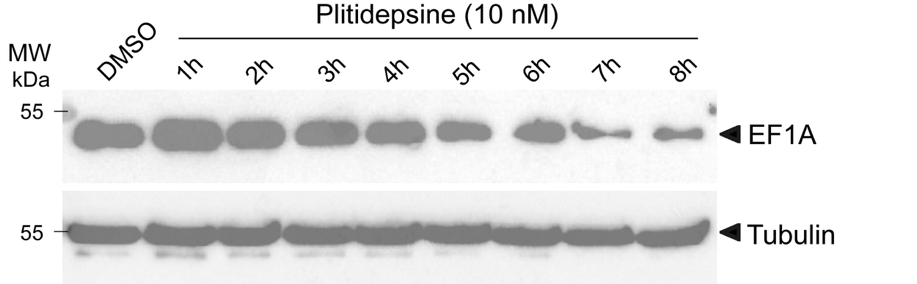
**

**Supplementary Figure 2. Kinetics of eEF1A degradation in BSRT7/5 cells incubated with 10 nM of plitidepsin.** BSRT7/5 cells were treated with serial dilutions of plitidepsin or DMSO for different times and the expression of eEF1A or tubulin were analyzed by Western blot.
